# Editorial for the Special Issue on Micro/Nano-Structure Based Optoelectronics and Photonics Devices

**DOI:** 10.3390/mi14101867

**Published:** 2023-09-29

**Authors:** Zhiyong Wu, Lu Zhu, Zhengji Xu

**Affiliations:** 1 School of Microelectronics Science and Technology, Sun Yat-sen University, Zhuhai 519082, China; wuzhy97@mail2.sysu.edu.cn; 2 Guangdong Provincial Key Laboratory of Optoelectronic Information Processing Chips and Systems, Sun Yat-sen University, Zhuhai 519082, China

In the ever-evolving fields of optoelectronics and photonics, the introduction of carefully designed micro-/nanostructures enables personalized customization of the electrical and optical properties of optoelectronic and photonic devices. As a result of the combination of micro-/nanomanufacturing technology and optoelectronics and photonics, desirable device performances are obtained, arousing many commercial potentials, including lighting, imaging, photovoltaics, optical communications, photoelectric detection, and biomedicine imaging [1,2,3,4,5]. Consequently, micro-/nanostructural design has become a research hotspot in the fields of optoelectronics and photonics. These micro-/nanostructures are fundamental building blocks of potential transformative optoelectronics and photonics devices and related systems, which can significantly increase their flexibility and applicability. This Special Issue showcases the valuable applications and advances of micro-/nanostructures in optoelectronic and photonic devices.

This Special Issue, entitled “Micro/Nano-Structure Based Optoelectronics and Photonics Devices,” includes some noteworthy studies: five research articles and one review article. The Special Issue covers a wide range of topics: near-infrared (NIR) liquid crystal multifunctional automated optical polarimeters [6], enhanced modulation bandwidth of distributed feedback (DFB) lasers [7], full-color reflective electrowetting displays (EWDs) [8], a three-port light flow controller enabled by a programmable multimode waveguide engine [9], a dye-sensitized solar cell (DSSC) electrolyte preparation method considering light path and light absorption [10], and a review of recent advances in electrically tunable lenses for imaging and light manipulation [11]. From the perspective of modulating and manipulating the polarization characteristics of light, Farrahi et al. [6] designed, calibrated, and developed a NIR liquid crystal multifunctional automated optical polarimeter, aiming to study and characterize the polarimetric properties of optical polymer nanofilms. Depending on the nanophotonic structure and composition of functionalized polymer nanomaterials, their optical properties can be tailored. In order to break the bottleneck of the carrier–photon resonance (CPR) frequency-limited bandwidth of the DFB directly modulated laser, Chi et al. [7] introduced the detuned photon–photon resonance (PPR) and restarted the CPR response by exploiting a time delay between the differential signals applied to the push–pull modulated DFB. Yang et al. [8] designed, fabricated, and measured a full-color reflective EWD system consisting of three layers of cyan, magenta, and yellow EWD elements. By adjusting the applied voltage, the aperture ratio, response time, and color gamut of the EWD element can be modified in real time. By utilizing microheaters on a waveguide chip to continuously adjust the local refractive index of the waveguide, Chen et al. [9] implemented an optical flow controller on a programmable multimode waveguide engine that can regulate three-port optical power in lossless and lossy modes. With just a simple waveguide structure and four microheaters, light can be freely routed to any of the three output ports at any power ratio, with or without additional attenuation. Furthermore, Yang et al. [10] studied the electrolyte formulation of a new DSSC with an external photoanode structure, which is one of the key components in DSSC. By configuring iodine-based electrolytes with a match between light absorption and light path, the photoelectric conversion capability of solar cells can be significantly adjusted. Finally, Chen et al. [11] reviewed the working principles and recent advances of electrowetting and dielectrophoretic electro-optical fluidic devices, including optical lenses/microscopes, beam control, and in-plane light manipulation, and discussed electromicrofluidic applications and some methods to improve lens performance.

In conclusion, this Special Issue reports on a variety of optoelectronic and photonic devices based on micro-/nanostructures, providing a window to understand the future prospects of optoelectronics and photonics. These results indicate that micro-/nanostructures are of great value for constructing novel optoelectronic and photonic devices or optimizing the working performance of existing optoelectronic and photonic devices. We predict that an increasing number of devices with micro-/nanostructures as basic building blocks will emerge in the future, greatly expanding the research fields of optoelectronics and photonics.
